# Percutaneous Transcatheter Aspiration of a Large Left Atrial Mass

**DOI:** 10.1016/j.jaccas.2024.102841

**Published:** 2024-12-18

**Authors:** Sumon Roy, YingWei Lum, Hamza Aziz, Rani K. Hasan

**Affiliations:** a Division of Cardiology, The Johns Hopkins University School of Medicine, Baltimore, Maryland, USA; b Division of Vascular Surgery and Endovascular Therapy, The Johns Hopkins University School of Medicine, Baltimore, Maryland, USA; c Division of Cardiac Surgery, The Johns Hopkins University School of Medicine, Baltimore, Maryland, USA

**Keywords:** aspiration, left atrial mass, percutaneous, thrombus, transcatheter

## Abstract

An 85-year-old woman with atrial fibrillation was found to have a large 4.5- × 3.5-cm left atrial mass. Transcatheter left atrial mass extraction was performed using an aspiration cannula, through which the bulk of the mass was retrieved. However, a fragment of the mass broke off and embolized distally. Right femoral arterial cutdown with distal thrombectomy was performed and a large chunk of the embolized mass was retrieved. Pathology of the specimen showed complex thrombus.

## Graphical Abstract

**Figure:**
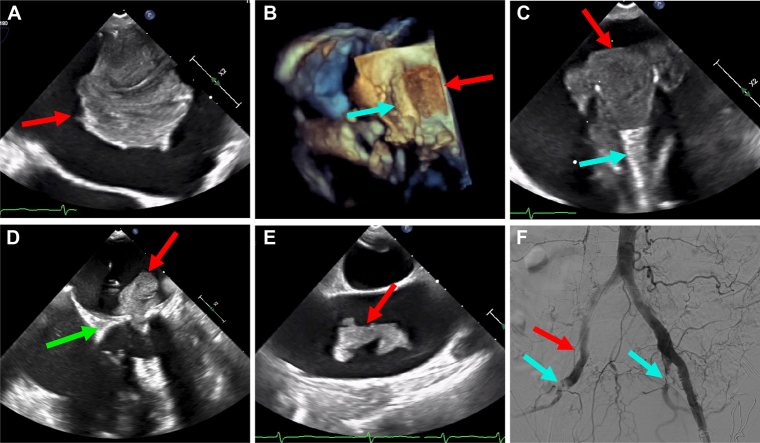

An 85-year-old woman with persistent atrial fibrillation (CHA_2_DS_2_-VASc score 5), mitral valve prolapse status post repair, recent percutaneous coronary intervention, and recurrent gastrointestinal bleeding presented for elective percutaneous transcatheter left atrial appendage occlusion (LAAO). Transesophageal echocardiography (TEE) was performed for procedural guidance, and preprocedural imaging revealed a large 4.5- × 3.5-cm LA mass attached to the posteromedial wall (Figure 1, Video 1). LAAO was aborted because the mass precluded LAAO device deployment together with concerns of tumor and/or complex thrombus. The patient was admitted for further work-up, computed tomography scan confirmed TEE findings, and the cardiothoracic surgery team deemed the patient high risk for surgical mass removal. She was therefore scheduled for transcatheter LA mass extraction using an aspiration cannula with bilateral cerebral embolic protection.

**Take-Home Message**

• Transcatheter retrieval is an option for percutaneous mass extraction in patients at high surgical risk.

**Figure 1:**
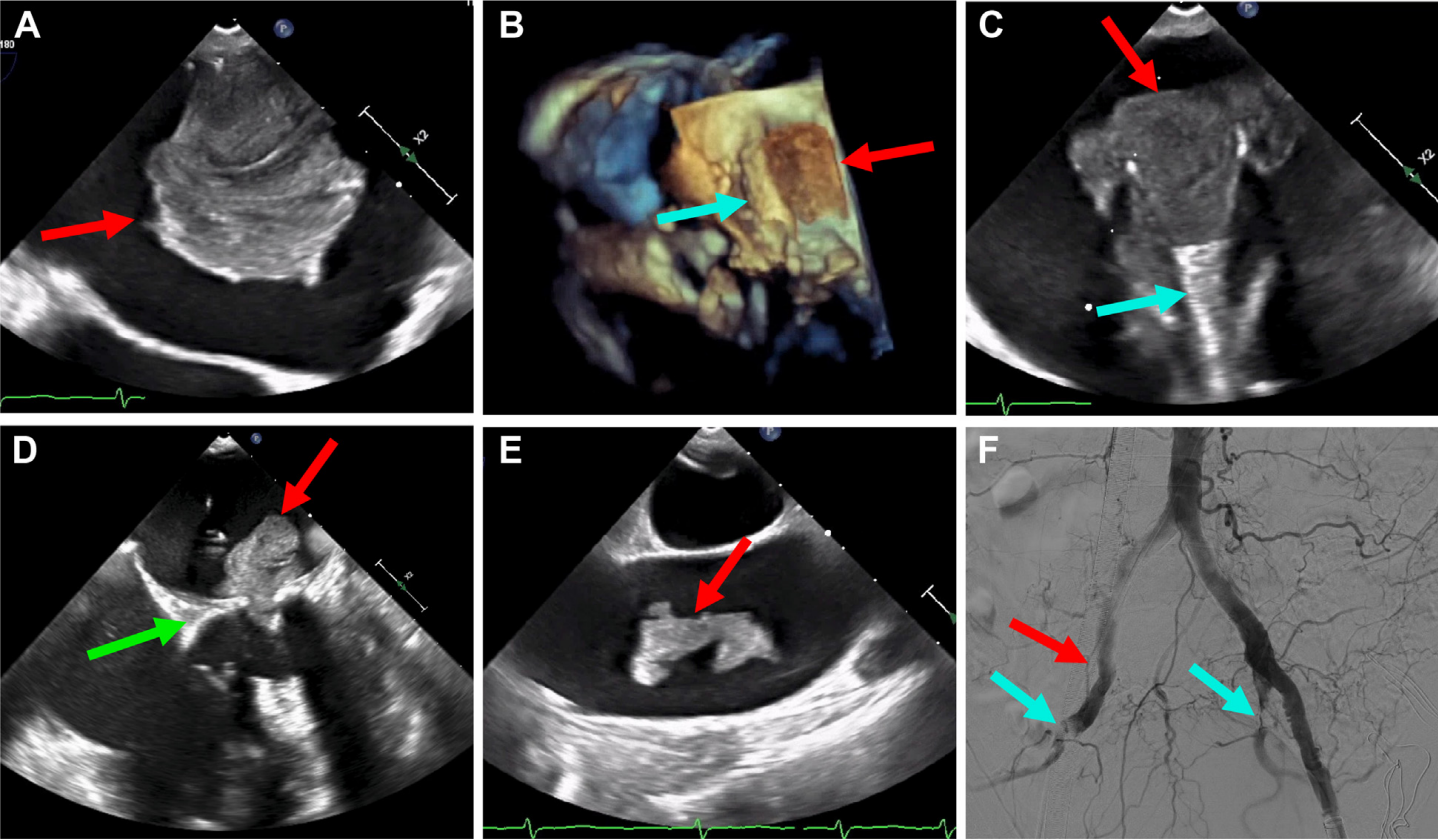
Transcatheter Aspiration of a Large Left Atrial Mass With an Embolized Fragment Occluding Peripheral Vasculature (A) Large left atrial (LA) mass (red arrow) as seen by transesophageal echocardiography (TEE). (B) Three-dimensional TEE showing large LA mass (red arrow). The aspiration catheter has been inserted into the left atrium (blue arrow) just adjacent to the LA mass. (C) The bulk of the LA mass (red arrow) being suctioned into the aspiration catheter (blue arrow). (D) A fragment of the LA mass (red arrow) broke off and is seen traversing the mitral valve (green arrow). (E) Embolized fragment of the LA mass (red arrow) in transit in the aortic arch. (F) Complete thrombotic occlusion of the ostial right external iliac artery (red arrow) and subtotal thrombotic occlusion of the proximal bilateral internal iliac arteries (blue arrows).

Embolic protection devices were deployed via the right transradial arterial access and left transfemoral arterial access, with the latter placed such that the distal filter was positioned in the proximal left subclavian artery and the proximal filter was positioned in the descending thoracic aorta, noting it would be undersized (left subclavian artery prohibitive for proximal filter). Transseptal puncture was performed via right transfemoral venous access, taking great care to navigate the sheath away from the LA mass with TEE guidance. Atrial septostomy was performed with a 14- × 40-mm balloon, which was then used to facilitate delivery of a 22-F AngioVac F-18 aspiration thrombectomy catheter. Using bilateral transfemoral access, suction was then applied to the system with flows through the circuit up to 3,000 rpm. The funnel of the aspiration cannula was externalized into the left atrium (Video 2) and then very slowly and cautiously oriented closer to and toward the mass to allow eventual suction of the bulk of the mass into the cannula (Video 3). However, a fragment of the mass broke off and embolized across the mitral valve into the left ventricle and exited the heart across the aortic valve, temporarily bobbing in the aortic arch (Video 4) before embolizing distally. TEE showed no remaining mass in the left atrium. Serial aortography demonstrated no evidence of thrombus or mass in the ascending aorta or arch vessels. There was also no evident thrombus in any of the embolic protection devices. The right external iliac artery was noted to be thrombotically occluded, and nonocclusive thrombus was identified in the bilateral internal iliac arteries (Video 5). There was also question of right renal artery and distal superior mesenteric artery compromise. Vascular surgery was emergently consulted, and aspiration with a Penumbra device was considered but deferred due to the possibility of further fragmentation and embolization. Therefore, right femoral arterial cutdown with distal thrombectomy was performed, and a large chunk of the embolized mass was retrieved. The patient was successfully extubated and taken to the cardiac intensive care unit in stable condition, neurologically intact with normal distal pulses. She was ultimately discharged on anticoagulation with plans to return for TEE-guided LAAO in 6 weeks. Pathology of the specimen showed multiple white-tan, irregular-shaped fragments of friable soft tissue (6.6 × 4.5 × 1.4 cm in aggregate) with adherent blood clot, most consistent with complex thrombus.

## Funding Support and Author Disclosures

The authors have reported that they have no relationships relevant to the contents of this paper to disclose.

## Appendix

Video 1 Large Left Atrial Mass as Seen by Transesophageal Echocardiography

Video 2 Three-Dimensional Rransesophageal Echocardiography Showing Large Left Atrial Mass The aspiration catheter has been inserted into the left atrium just adjacent to the left atrial mass

Video 3 Bulk of the Left Atrial Mass Being Suctioned Into the Aspiration Catheter

Video 4 Embolized Fragment of the Left Atrial Mass Bobbing in Transit in the Aortic Arch

Video 5 Complete Thrombotic Occlusion of the Ostial Right External Iliac Artery and Subtotal Thrombotic Occlusion of the Proximal Bilateral Internal Iliac Arteries as Seen by Digital Subtraction Angiography

